# Non-Target Arthropod Community Variation Associated with Abamectin Trunk-Injection History in a *Pinus massoniana* Plantation

**DOI:** 10.3390/biology15191744

**Published:** 2026-10-02

**Authors:** Xianheng Ouyang, Jinping Tian, Zhikuan Cao, Qiaoyun Sun

**Affiliations:** 1 College of Forestry, Northwest A&F University, Xianyang 712100, China; 2 College of Food Science and Engineering, Ocean University of China, Qingdao 266000, China; 3 College of Natural Resources and Environment, South China Agricultural University, Guangzhou 510642, China; 4 School of Architecture and Urban Planning, Shenzhen University, Shenzhen 518060, China

**Keywords:** pine wilt disease, abamectin, trunk injection, non-target arthropods, feeding guilds, community composition

## Abstract

Abamectin trunk injection is used to control pine wilt disease, but field evidence linking injection history to non-target arthropod communities is limited. We compared uninjected plots with plots injected once in March 2024 (ATI1) or March 2022 (ATI3) in a *Pinus massoniana* plantation in the Qinling–Daba Mountains. Three plots per history were sampled in July, September, and November 2025. Morphological identification and cytochrome c oxidase subunit I metabarcoding characterized soil-associated arthropods and aboveground Hymenoptera; feeding guilds were assigned at the family level. Litter residues and soil properties were measured. Overall Shannon diversity did not differ significantly among histories in either assemblage. Overall composition differed significantly for soil-associated arthropods but not Hymenoptera. Compositional differences occurred among soil detritivores, herbivores, and microbivores and among hymenopteran parasitoids, predators, and pollinators. Hymenopteran herbivore and pollinator diversity was higher in ATI1 and ATI3, respectively, whereas no soil feeding guild showed significant diversity differences. Residues were detected in both injected groups and were higher in ATI1. Redundancy analysis suggested directional associations with residues or soil properties, but these were descriptive rather than evidence of significant correlations. Injection-history associations were taxon- and metric-specific, supporting integrated diversity, composition, and feeding-guild assessments in non-target monitoring and risk evaluation.

## Simple Summary

Abamectin is injected into pine trunks to help control pine wilt disease, but its relationship with other arthropods living in these forests remains poorly understood. We compared Masson pine plots that had never been injected with plots that received a single injection in March 2024 or March 2022. Overall Shannon diversity, which combines the number of different taxa and the evenness of their representation, did not differ significantly among injection histories for either soil-associated arthropods or Hymenoptera, the insect group that includes bees, wasps, and ants. However, the overall composition of soil-associated arthropods differed among histories, whereas that of Hymenoptera did not. Differences also occurred within particular feeding groups: detritus feeders, plant feeders, and microbe feeders among soil-associated arthropods, and pollinators, predators, and parasitoids among Hymenoptera. Hymenopteran plant feeders had higher diversity in the more recently injected plots, whereas pollinators had higher diversity in the earlier-injected plots. These findings show that associations with injection history depend on the arthropod group and the measure examined. Evaluations of non-target organisms should therefore consider overall diversity, community composition, and variation within feeding groups together.

## 1. Introduction

Managing pine wilt disease requires balancing tree protection against the potential effects of chemical control on other organisms. The disease is caused by the pine wood nematode *Bursaphelenchus xylophilus* (Rhabditida: Aphelenchoididae), which is transmitted primarily by *Monochamus alternatus* (Coleoptera: Cerambycidae) in East Asia [1,2]. Masson pine, *Pinus massoniana* (Pinales: Pinaceae), is an important host in the Qinling–Daba Mountains of China, where the distribution and population structure of the insect vector are relevant to disease management [3]. Trunk injection delivers pesticides directly into trees. Abamectin belongs to the avermectin family of compounds and acts primarily on ligand-gated ion channels in invertebrates [4]. Throughout this study, “abamectin” denotes the active ingredient used in the field and is distinguished from both the broader class of avermectins and the different active ingredient emamectin benzoate.

Application method is central to non-target risk assessment because it influences when and where organisms encounter pesticides. Injecting a pesticide into the trunk creates exposure pathways that differ from foliar or soil application, but targeted delivery does not eliminate environmental exposure. In an apple orchard, Kou et al. [5] compared trunk injection and foliar application of thiamethoxam and found that application method influenced both pest control and non-target insect responses. Coslor et al. [6] showed that the timing of trunk injection affected subsequent pesticide residues in apple nectar and other plant tissues. Emamectin benzoate residues have also been detected in fallen leaves of injected horse chestnut trees [7]. These studies highlight the relevance of application method and plant-mediated exposure pathways, although their findings cannot be extrapolated quantitatively to abamectin in *P. massoniana* forests.

The choice of community metrics is equally important. Shannon diversity and community composition describe different aspects of community structure: similar overall Shannon diversity can coexist with differences in taxonomic membership or relative abundance. A field study of emamectin benzoate trunk injection, closely related to the present study, reported changes in soil arthropod composition despite limited changes in Shannon diversity [8]. Experiments in freshwater communities likewise show that pesticide-associated responses differ among structural and functional metrics [9]. Soil arthropods contribute to decomposition and nutrient cycling, whereas Hymenoptera include predators, parasitoids, and pollinators [10,11]. Examining whole assemblages alongside feeding guilds may therefore reveal differences obscured by a single overall diversity metric. Nevertheless, feeding-guild composition is not itself a direct measure of ecosystem functioning.

Environmental context further complicates the interpretation of field observations. For example, nutrient additions can modify soil arthropod responses during forest litter decomposition [12]. A European field study also found that pesticide exposure was associated with reduced bumble bee colony performance, with the relationship depending on landscape context [13]. Community differences associated with management history may thus reflect pesticide exposure, pre-existing environmental differences among plots, or both. Measurements of litter residues and soil properties help characterize this context but cannot, on their own, distinguish these explanations. DNA metabarcoding provides a complementary means of characterizing highly diverse arthropod communities, particularly when morphological identification is difficult [14]. However, sequence-based community profiles are not direct measurements of organismal abundance.

Here, we compared non-target arthropod communities among plots with different histories of abamectin trunk injection within a single *P. massoniana* plantation. Our objectives were to (1) compare overall Shannon diversity and community composition among injection histories; (2) determine whether associations differed among major taxonomic groups and feeding guilds; and (3) examine relationships between community characteristics, litter abamectin residues, and soil physicochemical properties. The findings provide field evidence relevant to the ecological risk assessment of trunk injection and the management of pine wilt disease.

## 2. Materials and Methods

### 2.1. Study Area, Plot Selection, and Injection History

The study was conducted in a continuous *P. massoniana* plantation in Langao County, Ankang, Shaanxi Province, China, within a study region spanning 32.05–32.50° N and 108.72–108.88° E. The plantation lies on the northern slope of the Daba Mountains, within the Qinling–Daba mountain region and a transition zone of the subtropical monsoon climate. Regional climatic context was drawn from the high-resolution temperature and precipitation dataset of Lu and Lu [15], which documents pronounced spatial climatic gradients across this mountain region. The soil is classified as mountain yellow-brown soil, and the understory includes *Itea*, *Melilotus*, and *Dryopteris*.

The plantation covered approximately 20 ha (300 mu) and was approximately 20 years old in 2025. No substantial thinning, fire, or other major disturbance had occurred during the preceding 10 years. Nine plots were selected to represent three injection histories: an uninjected control, a single injection in March 2024 (ATI1), and a single injection in March 2022 (ATI3), with three plots per history. Plots were selected to be as comparable as possible in stand age, tree density, canopy closure, slope, and aspect. Each plot covered approximately 0.33 ha (5 mu), giving a combined area of approximately 2.0 ha for the six injected plots. Each plot contained approximately 500 living *P. massoniana* trees, equivalent to approximately 1500 trees ha^−1^. Plots were separated by more than 100 m. This spacing reduced spatial proximity but did not establish statistical independence or prevent flying insects from moving among plots.

ATI1 and ATI3 denote more recent and earlier injection histories, respectively, rather than exactly 12 and 36 months after injection. At sampling in July–November 2025, approximately 16–20 months had elapsed since injection in ATI1 and 40–44 months in ATI3. Different plots represented the two histories; the study did not repeatedly observe the same plots before and after injection, and no pre-injection community data were available. The design was therefore an observational, space-for-time comparison in which treatment allocation and elapsed time could not be fully separated from pre-existing differences among plots.

Trunk injections used a commercial 5% (*w*/*w*) abamectin emulsifiable concentrate supplied in 20 mL bottles (pesticide registration number PD20170030; Shandong Baixin Biotechnology Co., Ltd., Heze, China). All living *P. massoniana* trees in the six injected plots were treated, totaling approximately 3000 trees; control plots were not injected. Formulation volume was determined by diameter at breast height (DBH): trees with DBH < 10 cm received one 20 mL bottle (20 mL tree^−1^); those with DBH of 10–20 cm received two bottles (40 mL tree^−1^); those with DBH of 20–30 cm received three bottles (60 mL tree^−1^); and those with DBH > 30 cm received four bottles (80 mL tree^−1^). The product concentration was specified as a mass fraction of 5% (*w*/*w*). Because field application was recorded by volume and formulation density was unavailable, the mass of active ingredient applied per tree could not be calculated reliably. The number of injection holes per tree equaled the number of bottles used. Bark was locally removed, holes were drilled with a dedicated tool, bottles were inserted into the holes, and injection quality was checked. No additional antifreeze was used. Each injected plot was treated only once, with no annual reapplication. Historical DBH records were unavailable for all individual trees, so identical DBH distributions among injection-history groups could not be confirmed. Nevertheless, plot selection sought to ensure broadly comparable stand age, tree density, and canopy structure.

### 2.2. Field Sampling and Sampling Units

Litter and soil were sampled in July, September, and November 2025. Five sampling points were established within each plot to capture within-plot spatial heterogeneity while retaining the plot as the spatial sampling unit. Surface litter was collected from a 20 × 20 cm quadrat at each point. After litter removal, the 0–5 cm soil layer was sampled using a corer 7 cm in diameter. For each plot and sampling occasion, litter and soil from the five points were pooled separately and thoroughly homogenized, producing one composite litter sample and one composite soil sample. The five sampling points were subsamples, not independent treatment replicates.

Three Malaise traps were installed in each plot, all more than 100 m from the forest edge. Traps operated continuously from July to November 2025, and collection bottles were replaced monthly. Samples from the three traps within each plot were pooled by month. Only samples collected in July, September, and November were included in the Hymenoptera analyses, yielding 27 plot-by-month samples from nine plots sampled in three months. For both assemblages, plots were the independent spatial sampling units. Repeated monthly sampling characterized temporal variation within plots but did not increase the number of independent plots. Each injection history was represented by three plot-level replicates.

### 2.3. Laboratory Analyses

#### 2.3.1. Arthropod Extraction and Identification

Small- and medium-sized arthropods with body widths <2 mm were extracted from litter and soil for 48 h using dry Tullgren funnels heated by 40 W incandescent lamps [16]. Extracted specimens were classified morphologically and counted under a stereomicroscope. For molecular analysis, aliquots of homogenized litter and soil were combined to produce a litter–soil composite for DNA extraction. Thus, the soil-associated arthropod assemblage considered here also includes litter-associated taxa. Malaise-trapped Hymenoptera were identified morphologically before molecular analysis.

#### 2.3.2. Litter Residues and Soil Physicochemical Properties

Abamectin residues in composite litter samples were measured by high-performance liquid chromatography (HPLC) using an Agilent 1100 system equipped with a Compass C18(2) reversed-phase column (250 × 4.6 mm, 5 μm). Samples were extracted twice with acetone, concentrated under nitrogen, and reconstituted in methanol. Detection was performed at 245 nm using methanol–water (90:10, *v*/*v*) as the mobile phase. Residue concentrations therefore represent abamectin in litter rather than mineral soil and are reported alongside soil properties in Table S1. Soil organic matter (OM) was determined by potassium dichromate oxidation with external heating; alkali-hydrolyzable nitrogen (AN) by alkali hydrolysis and diffusion; available phosphorus (AP) by colorimetry; available potassium (AK) by flame photometry; and pH by potentiometry, following Bao [17]. Residues not detected in control plots were recorded as zero in the summary table; this convention does not imply that their true concentrations were exactly zero. Abamectin residues are expressed as μg g^−1^ fresh weight (FW), OM as g kg^−1^, and AN, AK, and AP as mg kg^−1^; pH is dimensionless.

#### 2.3.3. DNA Extraction and Sequence Processing

Total DNA for soil-associated arthropod analysis was extracted from homogenized litter–soil composites using the DNeasy^®^ Blood & Tissue Kit (QIAGEN, Hilden, Germany), following the manufacturer’s instructions. To reduce differences in DNA input arising from body size, Hymenoptera were divided into individuals ≥0.5 cm and <0.5 cm in body length during tissue preparation only. Similar masses of thoracic muscle were taken from individuals ≥0.5 cm, whereas multiple smaller individuals were pooled because each provided insufficient tissue. Total DNA from pooled Hymenoptera samples was extracted using the phenol–chloroform method.

The mitochondrial cytochrome c oxidase subunit I (COI) region was amplified using primers LCO1490 and HCO2198 [18]. The PCR program comprised initial denaturation at 95 °C for 5 min; 35 cycles of 95 °C for 30 s, 50 °C for 30 s, and 72 °C for 40 s; and a final extension at 72 °C for 10 min. Libraries were sequenced on an Illumina MiSeq platform with 300 bp paired-end reads (PE300). Sequences were quality-filtered using Trimmomatic v0.41 [19] and merged using FLASH v1.2.11 [20]. Operational taxonomic units (OTUs) were clustered at 97% sequence similarity using USEARCH v10, and chimeric sequences were removed [21]. Representative sequences were taxonomically assigned using the RDP Classifier against a COI reference database with a confidence threshold of 0.7. Good’s coverage exceeded 97% for all samples.

### 2.4. Community Metrics and Statistical Analyses

Alpha diversity was calculated from OTU-level abundance tables. Shannon diversity was used to integrate taxonomic richness and evenness [22]. Taxonomic summaries for both soil-associated arthropods and Hymenoptera were compiled at the family level. Families with an overall relative sequence abundance <0.10% were excluded from feeding-guild assignments and subsequent guild-level analyses. Overall relative sequence abundance was defined as the number of sequences assigned to a family divided by the total number of sequences across all samples of the corresponding assemblage. Based on family-level feeding habits, soil-associated arthropods were assigned to microbivores, herbivores, predators, and detritivores, and Hymenoptera to parasitoids, predators, pollinators, and herbivores. Tables S3 and S5 provide guild assignments and supporting references for the retained families; Tables S2 and S4 provide complete taxonomic summaries and relative abundances.

Differences among injection histories in Shannon diversity and the environmental variables listed in Table S1 were assessed using one-way analysis of variance (ANOVA), followed by least significant difference (LSD) post hoc comparisons. The mean of the three monthly observations for each plot was used as the replicate value (*n* = 3 plots per history). Data are expressed as means ± standard deviations (SDs); different lowercase letters indicate significant differences at *p* < 0.05.

Shannon diversity, principal coordinates analysis (PCoA), analysis of similarities (ANOSIM), and redundancy analysis (RDA) were implemented using vegan v2.6-4. PCoA based on Bray–Curtis dissimilarities visualized differences in OTU composition for whole assemblages and individual feeding guilds, and ANOSIM was used to compare groups. RDA described relationships between communities, litter abamectin residues, and the five soil properties. Response matrices were Hellinger-transformed before RDA.

## 3. Results

### 3.1. Litter Residues and Soil Properties

Litter abamectin residues differed among injection histories. Mean concentrations were 0.34 ± 0.03 μg g^−1^ FW in ATI1 and 0.08 ± 0.09 μg g^−1^ FW in ATI3, whereas residues were not detected in controls (means ± SDs; Table S1). These values reflect a comparison among different plots sampled in 2025, not repeated residue measurements from the same treated trees. Soil OM, AN, pH, AK, and AP did not differ significantly among injection histories (Table S1).

### 3.2. Taxonomic and Feeding-Guild Composition

**Figure 1:**
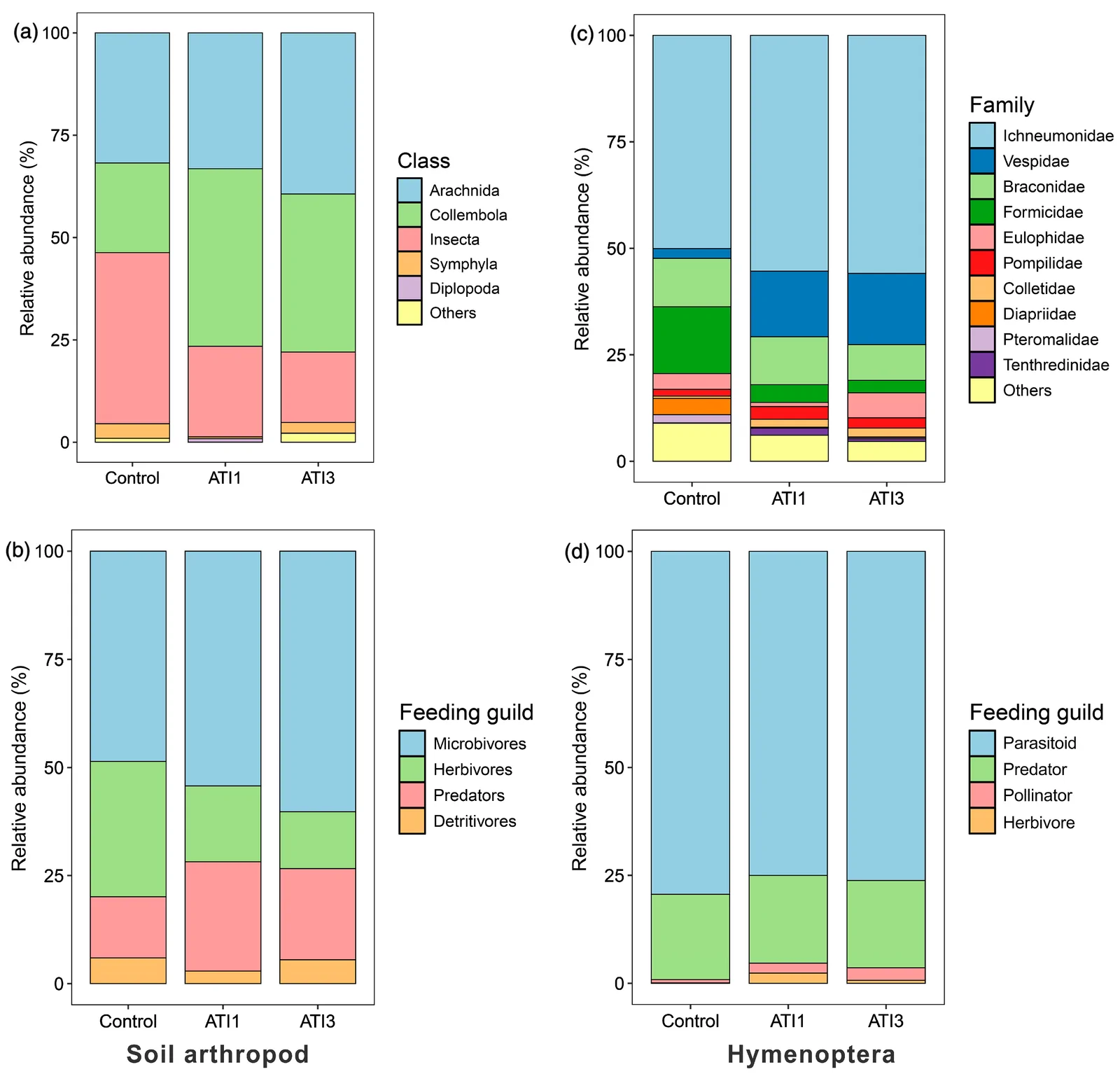
Relative sequence composition of arthropod taxonomic groups and feeding guilds across abamectin trunk-injection histories. (**a**) The five most abundant classes of soil-associated arthropods, with the remaining taxa grouped as “Others”; (**b**) soil-associated arthropod feeding guilds; (**c**) the ten most abundant hymenopteran families, with the remaining taxa grouped as “Others”; and (**d**) hymenopteran feeding guilds. Control, uninjected plots; ATI1, plots receiving a single abamectin trunk injection in March 2024; ATI3, plots receiving a single injection in March 2022. Feeding guilds are operational assignments based on family-level feeding habits (Tables S3 and S5), not direct measurements of ecosystem functioning.

The soil-associated arthropod dataset comprised 197,586 quality-filtered sequences assigned to 690 OTUs (Table S2). Arachnida, Collembola, and Insecta accounted for 34.86%, 34.47%, and 27.07% of sequence abundance, respectively. Microbivores represented the largest share of the assigned soil feeding guilds (Figure 1a,b; Table S3).

The Hymenoptera dataset comprised 378 OTUs across the three injection histories (Table S4). Ichneumonidae, Vespidae, and Braconidae accounted for 53.67%, 11.53%, and 10.34% of relative sequence abundance, respectively. Twenty-six named families with relative abundances above 0.10% were included in the feeding-guild summaries (Table S5). Parasitoids were the dominant guild, followed by predators, with pollinators and herbivores accounting for smaller proportions (Figure 1c,d).

### 3.3. Shannon Diversity

**Figure 2:**
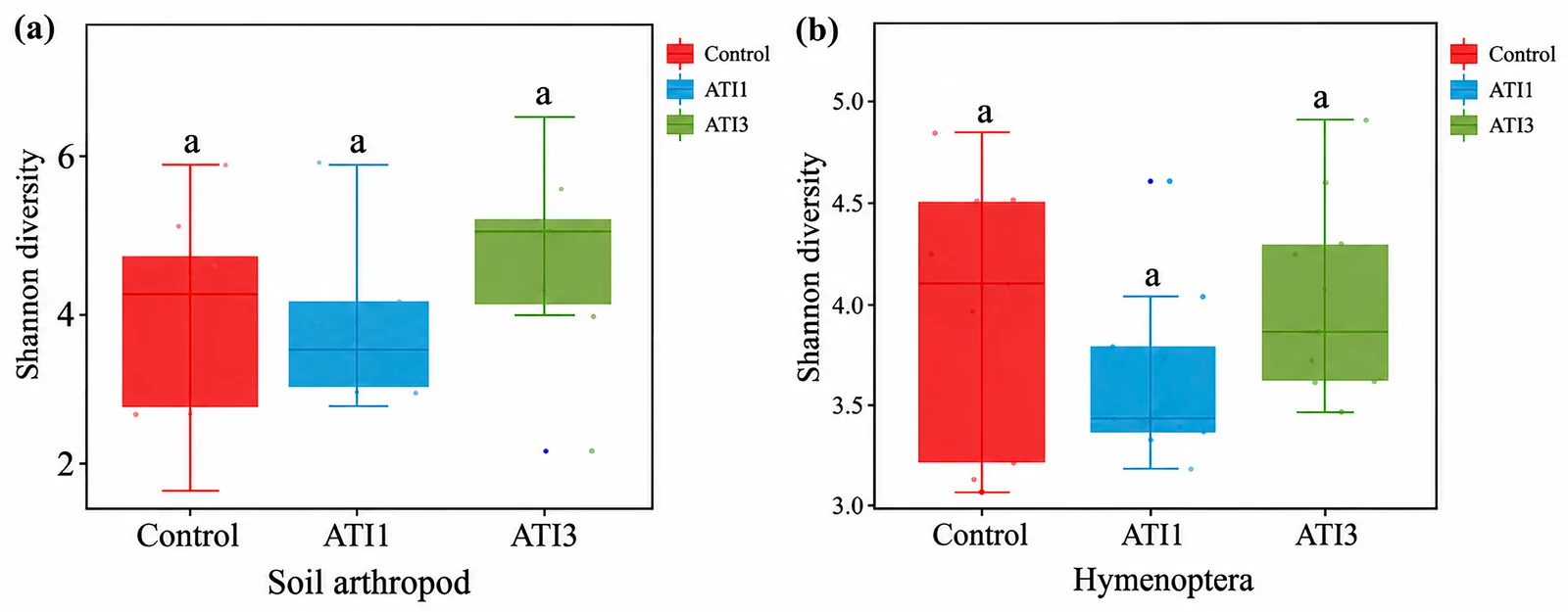
Shannon diversity of arthropod communities across abamectin trunk-injection histories. (**a**) Soil-associated arthropods; (**b**) Hymenoptera. Control, uninjected plots; ATI1, a single injection in March 2024; ATI3, a single injection in March 2022. Comparisons among injection histories used LSD post hoc tests. Identical lowercase letters indicate no significant difference (*p* > 0.05), whereas different letters indicate significant differences (*p* < 0.05).

Overall Shannon diversity did not differ significantly among the three injection histories for either soil-associated arthropods or Hymenoptera (Figure 2; *p* > 0.05). Similarly, none of the four soil-associated feeding guilds—detritivores, herbivores, microbivores, and predators—differed significantly in Shannon diversity among Control, ATI1, and ATI3 (Figure S1; *p* > 0.05).

In contrast, hymenopteran feeding guilds showed distinct associations with injection history (Figure S2). Herbivore Shannon diversity was significantly higher in ATI1 than in Control and ATI3, which did not differ from each other. Pollinator Shannon diversity was significantly higher in ATI3 than in Control and ATI1, with no significant difference between the latter two groups. Predator and parasitoid Shannon diversity did not differ significantly among the three histories (*p* > 0.05).

### 3.4. Differences in Community Composition

**Figure 3:**
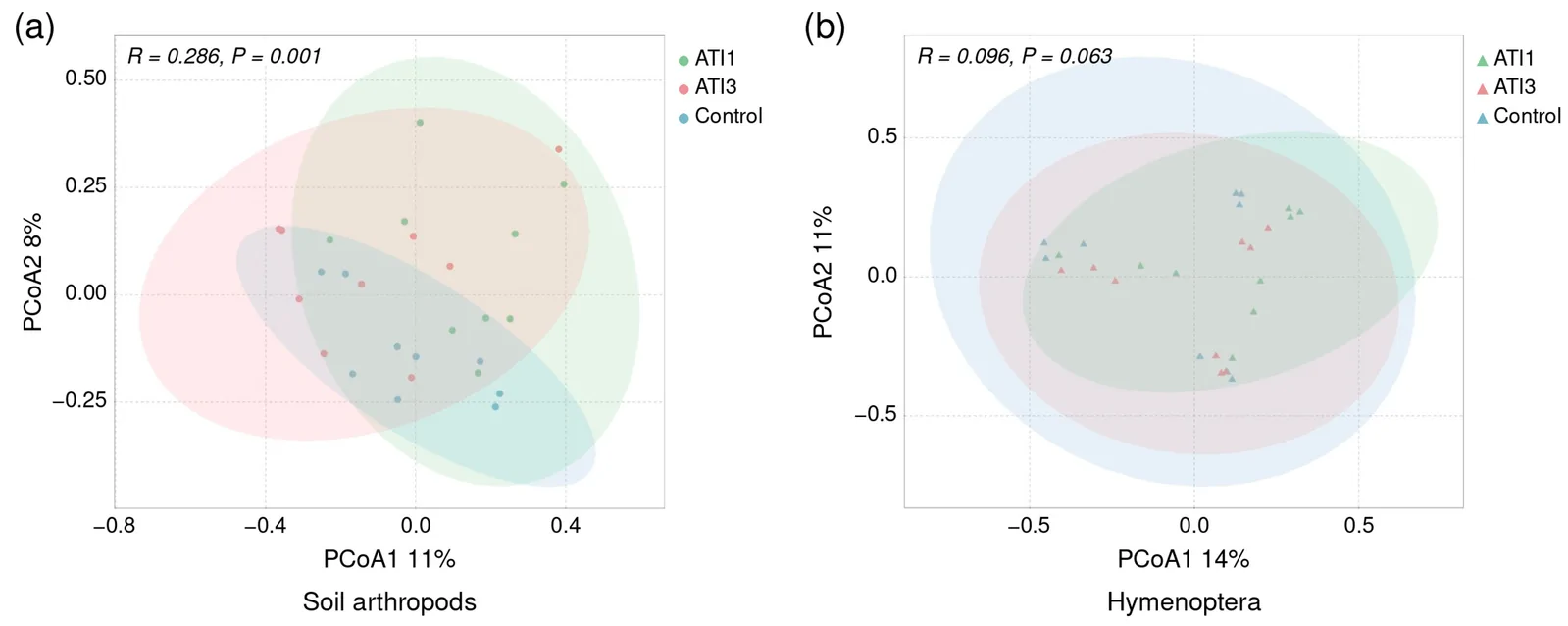
Principal coordinates analysis (PCoA) of OTU composition for (**a**) soil-associated arthropods and (**b**) Hymenoptera, based on Bray–Curtis dissimilarities. Points represent plot-by-month observations; repeated months within the same plot are not additional independent treatment replicates. Each injection history comprises three plots. Control, uninjected plots; ATI1, a single injection in March 2024; ATI3, a single injection in March 2022. Ellipses indicate 95% confidence regions for each group. ANOSIM *R* statistics and *p* values are shown in each panel, and the variance explained by each PCoA axis is given in parentheses.

The first two PCoA axes together explained 19% of the variation in soil-associated arthropod composition and 25% in Hymenoptera composition. Samples from different injection histories overlapped in ordination space for both assemblages. Nevertheless, overall soil-associated arthropod composition differed significantly among histories (ANOSIM, *R* = 0.286, *p* = 0.001; Figure 3a), whereas separation among the overall Hymenoptera assemblages was weaker and not significant (Figure 3b). Guild-level analyses indicated differences among injection histories for soil detritivores, herbivores, and microbivores, while soil predators showed greater overlap. Among Hymenoptera, pollinators, predators, and parasitoids differed among histories, whereas herbivores did not show clear differences (Figures S3 and S4). Overall, compositional differentiation associated with injection history was more pronounced in soil-associated arthropods than in Hymenoptera.

### 3.5. Descriptive Community–Environment Ordination

**Figure 4:**
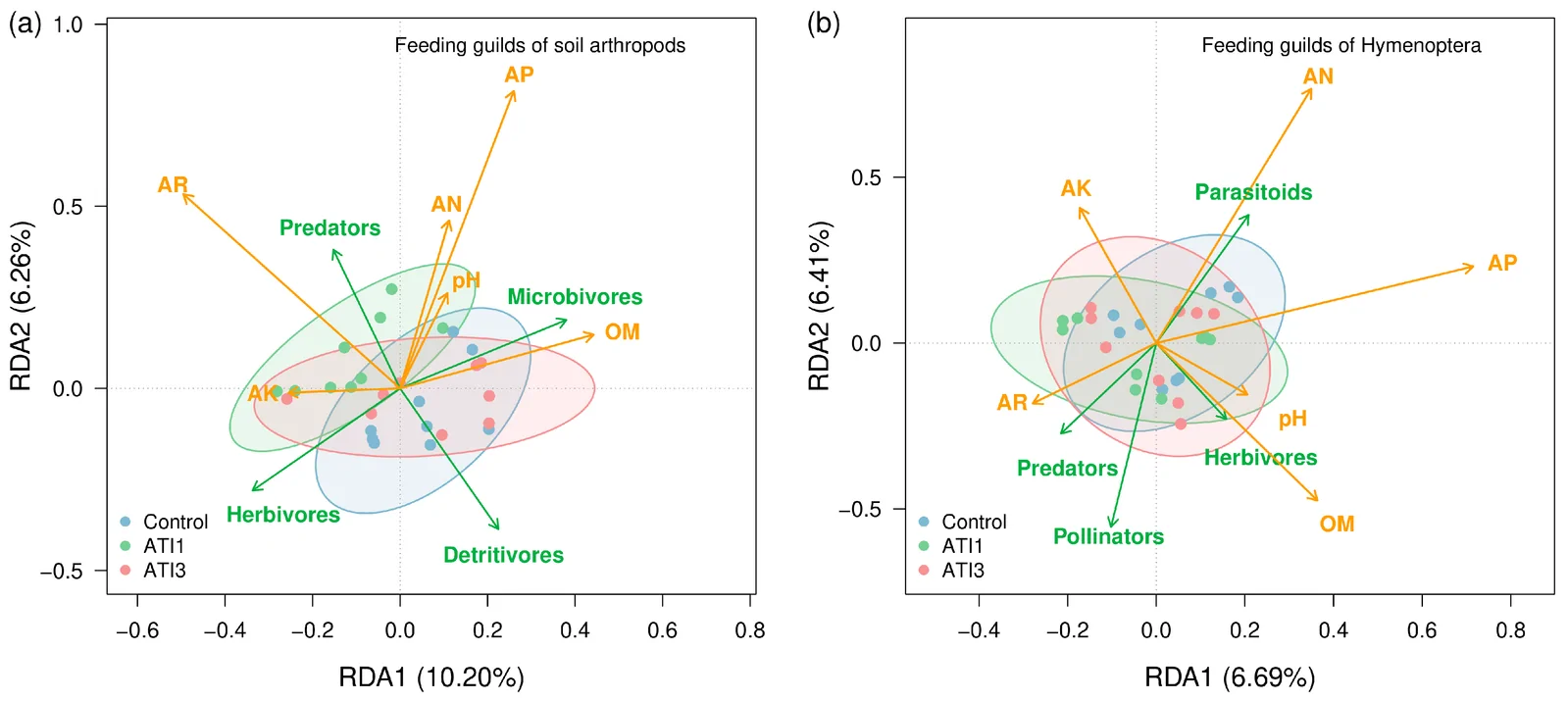
Redundancy analysis (RDA) ordinations for (**a**) soil-associated arthropods and (**b**) Hymenoptera. Orange vectors represent litter abamectin residues and soil properties; green vectors represent feeding guilds. AR, litter abamectin residues; AN, alkali-hydrolyzable nitrogen; OM, organic matter; AK, available potassium; AP, available phosphorus. Control, uninjected plots; ATI1, a single injection in March 2024; ATI3, a single injection in March 2022. Points represent plot-by-month observations rather than independent treatment replicates. Axis labels indicate the corresponding percentages explained. Vector directions and group overlap are descriptive and do not establish statistical significance, causality, or the independent contribution of any environmental variable.

The first two RDA axes together accounted for 16.46% and 13.10% in the soil-associated arthropod and Hymenoptera ordinations, respectively (Figure 4). In the soil-associated arthropod ordination, the microbivore vector was broadly aligned with OM, and the predator vector with litter abamectin residues (AR). In the Hymenoptera ordination, parasitoids were broadly aligned with AN, predators with AR, and herbivores with pH and OM. Injection-history groups overlapped substantially in both ordinations. These directional relationships are descriptive and do not demonstrate significant correlations.

## 4. Discussion

### 4.1. Overall Diversity and Composition Capture Different Community Responses

Overall Shannon diversity did not differ detectably among injection histories for either soil-associated arthropods or Hymenoptera, but their compositional patterns differed: significant differences occurred in the overall soil-associated assemblage, whereas those in Hymenoptera were not significant. Similar Shannon diversity therefore does not imply identical taxonomic membership or relative sequence representation. A study of emamectin benzoate trunk injection likewise reported contrasting patterns in overall diversity and community composition [8], and research on forestry insecticides has shown heterogeneous compositional responses among taxonomic groups [23]. Together, these findings support combining diversity and compositional metrics in non-target assessments. However, differences in active ingredients and exposure routes preclude using those studies to infer the magnitude of abamectin effects here.

Compositional differences among soil-associated arthropods occurred mainly within detritivores, microbivores, and herbivores, although Shannon diversity did not differ significantly in any of the four guilds. Separation among histories was relatively greater for microbivores, whereas the smaller *R* value for herbivores indicates that a statistically detectable difference need not correspond to pronounced community separation. A meta-analysis by Beaumelle et al. [24] showed that soil faunal responses to pesticides vary with exposure context and taxonomic group. Evidence of pesticide sensitivity in Collembola likewise supports examining particular taxa in addition to overall metrics [25]. The present findings should therefore be interpreted primarily as differences in OTU composition within feeding guilds.

Hymenopteran guilds showed different patterns in diversity and composition. Herbivore diversity was higher in ATI1 and pollinator diversity in ATI3, whereas neither parasitoids nor predators differed significantly in Shannon diversity. Conversely, compositional differences were detected among parasitoids, predators, and pollinators but not herbivores. This contrast underscores the need to interpret within-guild diversity and taxonomic composition separately. Separation among histories was limited for pollinators and predators and should not be described as strong or consistent community replacement. Although pesticide research has documented diverse non-target responses in natural enemies and pollinators [26,27,28], the present results concern differences in sequence composition and diversity alone.

### 4.2. Litter Residues and Community–Environment Relationships

Abamectin residues were detected in litter from both ATI1 and ATI3, with higher concentrations in ATI1, indicating that litter should be included in environmental assessments of trunk injection. Studies in other tree species have shown that application timing affects the distribution of residues within plant tissues [6] and that emamectin benzoate can occur in fallen leaves of injected horse chestnut trees [7]. Residue detection, however, is not equivalent to biologically available exposure, which also depends on binding within plant material, release processes, and routes of contact with animals. Toxicological studies of avermectins provide grounds for examining non-target soil fauna [29], but toxicity thresholds established for other compounds or experimental conditions cannot be directly compared with the litter concentrations measured here. Without residue measurements in mineral soil or animal tissues, actual exposure doses for the different feeding guilds cannot be quantified.

In the RDA ordinations, soil microbivores were broadly aligned with OM and soil predators with AR; hymenopteran parasitoids were aligned with AN, predators with AR, and herbivores with pH and OM. These patterns may inform hypotheses about resource conditions and exposure context, but they do not demonstrate that residues promote predators or establish any variable as a principal driver. The first two axes together accounted for 16.46% and 13.10% in the respective ordinations, and the injection-history groups overlapped considerably. A European multisite study similarly found taxon- and feeding-guild-specific associations between pesticide residues and soil biota that required interpretation in their environmental context [30]. The absence of significant differences in the measured soil properties does not exclude variation in unmeasured factors, such as litter quality, microclimate, vegetation, or food resources. For Malaise-trapped Hymenoptera in particular, associations with soil variables may reflect plot environmental conditions only indirectly.

### 4.3. Limits of Inference and Implications for Non-Target Monitoring

Different plots represented the injection histories, no pre-injection baseline was available, and each history was represented by only three plots. Consequently, injection timing could not be fully separated from pre-existing plot differences [31]. Lower residues or higher diversity in particular guilds in ATI3 do not establish a dissipation rate, community recovery, or compensatory response. Hymenoptera may also move among plots, and their capture composition can depend on surrounding resources and seasonal activity. Research on European bumble bees has shown that landscape context and food resources can modify performance associated with pesticide exposure [13]. Repeated monthly sampling improved seasonal coverage but did not increase the number of independent plots. Moreover, ANOSIM can reflect differences in within-group dispersion rather than only shifts in group centroids [32]. The present findings are therefore best regarded as exploratory evidence for subsequent testing.

Metabarcoding and family-level guild assignments further constrain interpretation. OTUs are not confirmed species, and sequence proportions are influenced by sampling, DNA extraction, and amplification biases; they cannot be equated directly with numbers of individuals or biomass [14,33]. Family-level feeding assignments do not fully capture differences among life stages, omnivory, or variation within families, and the 0.10% filtering threshold may exclude rare taxa. Future monitoring should therefore report both diversity and composition for whole assemblages and major feeding guilds, while combining chemical residues with direct measures of organismal exposure. Studies with more independent stands and plots, continuous observations before and after injection, and analyses that retain plots as treatment replicates would allow the observed associations and their ecological significance to be tested more rigorously.

## 5. Conclusions

In this *P. massoniana* plantation, overall Shannon diversity did not differ significantly among injection histories for either soil-associated arthropods or Hymenoptera. Nevertheless, overall soil-associated arthropod composition differed among histories, and particular feeding guilds in both assemblages showed history-associated variation; overall Hymenoptera composition did not differ significantly. Hymenopteran herbivore and pollinator diversity was higher in ATI1 and ATI3, respectively, while litter residues were higher in ATI1. These findings indicate that patterns associated with trunk injection are taxon- and metric-specific. Non-target assessments should integrate overall diversity, community composition, major feeding guilds, and exposure measurements rather than infer ecological safety or recovery from any single indicator.

## Data Availability

The original contributions presented in this study are included in the article/Supplementary Materials. Further inquiries can be directed to the corresponding author.

## Supplementary Materials

The following supporting information can be downloaded at: https://www.mdpi.com/article/10.3390/biology15191744/s1, Figure S1: Shannon diversity of soil-associated arthropod feeding guilds; Figure S2: Shannon diversity of hymenopteran feeding guilds; Figure S3: PCoA of soil-associated arthropod feeding guilds; Figure S4: PCoA of hymenopteran feeding guilds; Table S1: Litter abamectin residues and soil physicochemical properties; Table S2: Taxonomic composition of soil-associated arthropods; Table S3: Family-level feeding-guild assignments for soil-associated arthropods; Table S4: Family-level composition of Hymenoptera; Table S5: Family-level feeding-guild assignments for Hymenoptera. References [34,35,36,37,38,39,40,41,42,43,44,45,46,47,48,49,50,51,52,53,54,55,56,57,58,59,60,61,62,63,64,65,66,67,68,69,70,71,72,73,74,75,76,77,78,79,80,81,82,83,84,85,86,87,88,89,90,91,92,93,94,95,96,97,98,99,100,101,102,103,104,105] are cited in the Supplementary Materials.
