# Supplementary material for: Non-Target Arthropod Community Variation Associated with Abamectin Trunk-Injection History in a Pinus massoniana Plantation
Source: Biology (Basel). 2026 Oct 2;15(19):1744. doi: 10.3390/biology15191744 (PMC13663433; doi:10.3390/biology15191744)
Supplement: Supplementary file 1 [file biology-15-01744-s001.zip › biology-4550696-supplementary.pdf]

Supplementary Materials
Non-Target Arthropod Community Variation Associated with Abamectin Trunk-Injection History in a *Pinus massoniana* Plantation

Xianheng Ouyang [1], Jinping Tian [2], Zhikuan Cao [3] and Qiaoyun Sun [4,*]

[1] College of Forestry, Northwest A&F University, Xianyang 712100, China
[2] College of Food Science and Engineering, Ocean University of China, Qingdao 266000, China
[3] College of Natural Resources and Environment, South China Agricultural University, Guangzhou 510642, China
[4] School of Architecture and Urban Planning, Shenzhen University, Shenzhen 518060, China
* Correspondence: qiaoyun@szu.edu.cn

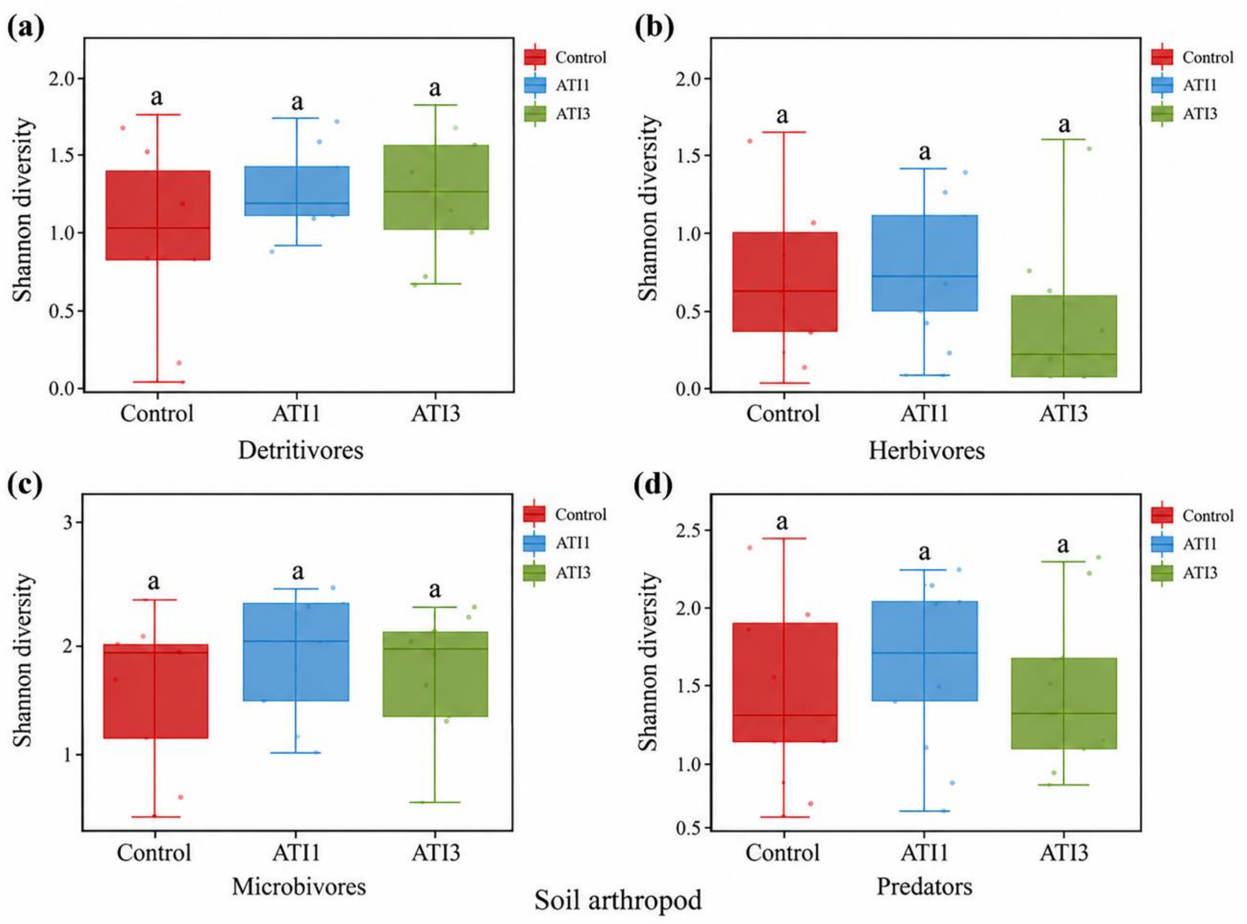

Figure S1. Shannon diversity of soil-associated arthropod feeding guilds across abamectin trunk-injection histories. (a) Detritivores; (b) herbivores; (c) microbivores; (d) predators. Control, uninjected plots; ATI1, a single injection in March 2024; ATI3, a single injection in March 2022. Shannon diversity was compared among injection histories using least significant difference (LSD) post hoc tests. Identical lowercase letters indicate no significant difference ($p > 0.05$); different letters indicate a significant difference ($p < 0.05$).

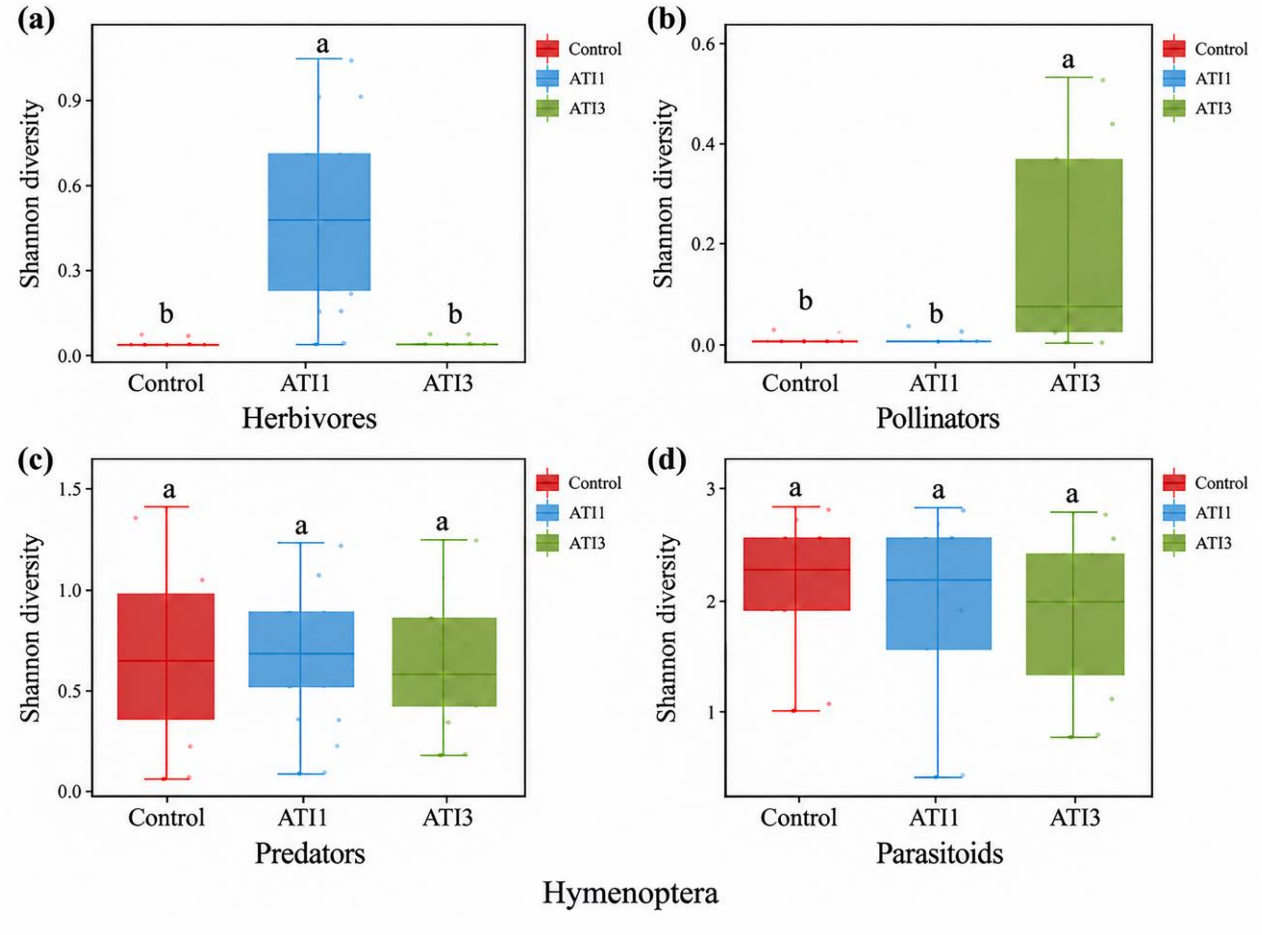

Figure S2. Shannon diversity of hymenopteran feeding guilds across abamectin trunk-injection histories. (a) Herbivores; (b) pollinators; (c) predators; (d) parasitoids. Control, uninjected plots; ATI1, a single injection in March 2024; ATI3, a single injection in March 2022. Shannon diversity was compared among injection histories using least significant difference (LSD) post hoc tests. Identical lowercase letters indicate no significant difference ($p > 0.05$); different letters indicate a significant difference ($p < 0.05$).

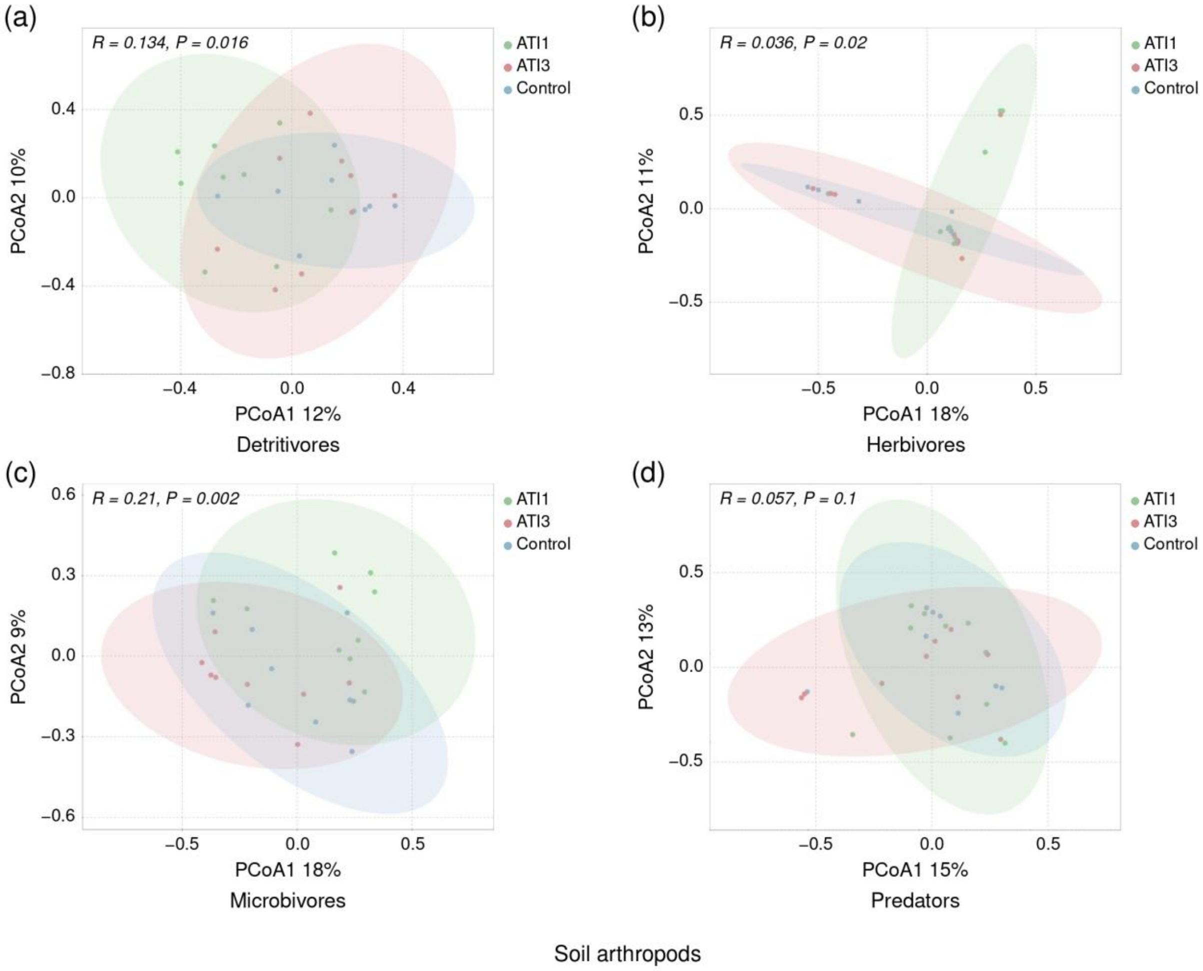

Figure S3. Principal coordinates analysis (PCoA) of OTU composition within soil-associated arthropod feeding guilds. (a) Detritivores; (b) herbivores; (c) microbivores; (d) predators. Points represent plot-by-month observations; repeated months within the same plot are not additional independent treatment replicates. Each injection history comprises three plots. Control, uninjected plots; ATI1, a single injection in March 2024; ATI3, a single injection in March 2022. Ellipses indicate 95% confidence regions for each group.

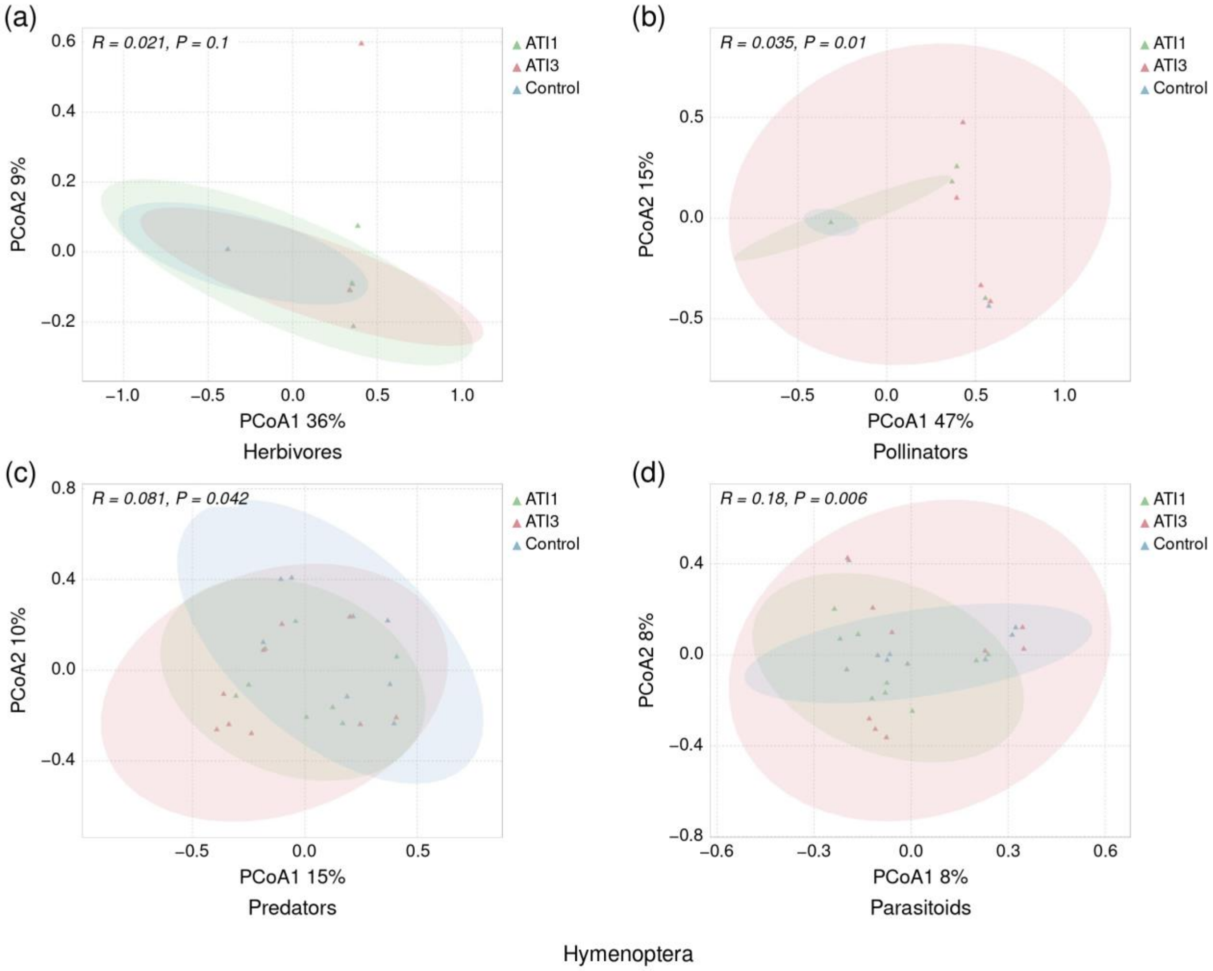

Figure S4. Principal coordinates analysis (PCoA) of OTU composition within hymenopteran feeding guilds. (a) Herbivores; (b) pollinators; (c) predators; (d) parasitoids. Points represent plot-by-month observations; repeated months within the same plot are not additional independent treatment replicates. Each injection history comprises three plots. Control, uninjected plots; ATI1, a single injection in March 2024; ATI3, a single injection in March 2022. Ellipses indicate 95% confidence regions for each group.

Table S1. Litter abamectin residues and soil physicochemical properties across abamectin trunk-injection histories.

Injection history	Litter abamectin (μg g⁻¹ FW)	Organic matter (g kg⁻¹)	Alkali-hydrolyzable nitrogen (mg kg⁻¹)	pH	Available potassium (mg kg⁻¹)	Available phosphorus (mg kg⁻¹)
ATI3 (March 2022)	0.08 ± 0.09 [b]	67.34 ± 16.52 [ns]	85.55 ± 8.76 [ns]	4.87 ± 0.29 [ns]	113.22 ± 6.45 [ns]	1.53 ± 0.65 [ns]
ATI1 (March 2024)	0.34 ± 0.03 [a]	66.58 ± 15.89 [ns]	85.04 ± 8.21 [ns]	5.07 ± 0.13 [ns]	116.22 ± 9.83 [ns]	2.66 ± 1.12 [ns]
Control (uninjected)	0.00 ± 0.00 [c]	59.17 ± 13.47 [ns]	88.09 ± 15.23 [ns]	4.91 ± 0.25 [ns]	114.56 ± 17.31 [ns]	2.16 ± 1.24 [ns]

Data are means ± standard deviations. Different lowercase letters indicate significant differences ($p < 0.05$); ns indicates no significant difference ($p \geq 0.05$). Control, uninjected plots; ATI1, a single injection in March 2024; ATI3, a single injection in March 2022. Abamectin residues were measured in composite litter samples, not mineral soil. FW, fresh weight.

Table S2. Taxonomic composition, numbers of operational taxonomic units (OTUs), and relative sequence abundance of soil-associated arthropods.

Phylum	Class	Order	Family	OTUs	Sequence reads	Relative sequence abundance (%)
**Arthropoda**				**690**	**197586**	**100.00%**
	**Arachnida**			**269**	**68873**	**34.86%**
		**Araneae**		**21**	**6555**	**3.32%**
			Anyphaenidae	1	8	0.00%
			Cheiracanthiidae	1	46	0.02%
			Linyphiidae	5	3981	2.01%
			Mysmenidae	2	182	0.09%
			Salticidae	4	1025	0.52%
			Selenopidae	1	1	0.00%
			Tetragnathidae	1	552	0.28%
			Theridiidae	1	128	0.06%
			Thomisidae	2	5	0.00%
			unclassified_Araneae	1	84	0.04%
			Zodariidae	2	543	0.27%
		**Mesostigmata**		**57**	**15776**	**7.98%**
			Ascidae	14	6710	3.40%
			Digamasellidae	1	10	0.01%
			Laelapidae	3	15	0.01%
			Macrochelidae	1	449	0.23%
			Melicharidae	2	473	0.24%
			Ologamasidae	2	228	0.12%
			Pachylaelapidae	1	103	0.05%
			Parasitidae	9	4690	2.37%
			Parholaspididae	3	2585	1.31%

Phylum	Class	Order	Family	OTUs	Sequence reads	Relative sequence abundance (%)
			Phytoseiidae	7	120	0.06%
			Polyaspididae	1	55	0.03%
			Rhodacaridae	4	104	0.05%
			unclassified_Mesostigmata	3	8	0.00%
			Uropodidae	2	56	0.03%
			Veigaiidae	2	42	0.02%
			Zerconidae	2	128	0.06%
		**Pseudoscorpiones**		**6**	**3260**	**1.65%**
			Garypinidae	1	429	0.22%
			Neobisiidae	5	2831	1.43%
		**Sarcoptiformes**		**117**	**35200**	**17.82%**
			Achipteriidae	2	1893	0.96%
			Alycidae	3	32	0.02%
			Anderemaeidae	1	52	0.03%
			Brachychthoniidae	10	282	0.14%
			Camisiidae	9	3741	1.89%
			Cepheidae	1	15	0.01%
			Ceratozetidae	10	835	0.42%
			Chamobatidae	3	25	0.01%
			Damaeidae	1	15	0.01%
			Eremaeidae	5	193	0.10%
			Euphthiracaridae	1	40	0.02%
			Fortuyniidae	2	626	0.32%
			Galumnidae	4	452	0.23%
			Haplozetidae	1	27	0.01%

Phylum	Class	Order	Family	OTUs	Sequence reads	Relative sequence abundance (%)
			Hermanniellidae	1	7	0.00%
			Hydrozetidae	1	12	0.01%
			Hypochthoniidae	3	22818	11.55%
			Malaconothridae	7	164	0.08%
			Micreremidae	1	16	0.01%
			Nanorchestidae	7	59	0.03%
			Nothridae	2	412	0.21%
			Oppiidae	9	408	0.21%
			Oribatellidae	2	9	0.00%
			Phenopelopidae	4	38	0.02%
			Scheloribatidae	3	2402	1.22%
			Scutoverticidae	2	17	0.01%
			Selenoribatidae	1	49	0.02%
			Steganacaridae	1	4	0.00%
			Suctobelbidae	5	10	0.01%
			Tectocepheidae	4	32	0.02%
			Tegoribatidae	1	2	0.00%
			Trhypochthoniidae	6	344	0.17%
			unclassified_Sarcoptiformes	3	26	0.01%
			Zetomimidae	1	143	0.07%
		**Trombidiformes**		**60**	**8059**	**4.08%**
			Anystidae	2	175	0.09%
			Bdellidae	11	1117	0.57%
			Erythraeidae	3	14	0.01%
			Eupodidae	18	471	0.24%

Phylum	Class	Order	Family	OTUs	Sequence reads	Relative sequence abundance (%)
			Iolinidae	1	3	0.00%
			Rhagidiidae	8	5495	2.78%
			Scutacaridae	1	6	0.00%
			Smarididae	1	4	0.00%
			Stigmaeidae	3	356	0.18%
			Triophtydeidae	1	1	0.00%
			Trombidiidae	1	38	0.02%
			Tydeidae	3	124	0.06%
			unclassified_Trombidiformes	7	255	0.13%
		**unclassified_Arachnida**		**8**	**23**	**0.01%**
			unclassified_Arachnida	8	23	0.01%
	**Chilopoda**			**16**	**180**	**0.09%**
		**Lithobiomorpha**		**16**	**180**	**0.09%**
			Lithobiidae	16	180	0.09%
	**Collembola**			**151**	**68110**	**34.47%**
		**Entomobryomorpha**		**82**	**39221**	**19.85%**
			Entomobryidae	50	11676	5.91%
			Isotomidae	26	23439	11.86%
			Paronellidae	1	2	0.00%
			Tomoceridae	5	4104	2.08%
		**Neelipleona**		**2**	**35**	**0.02%**
			Neelidae	2	35	0.02%
		**Poduromorpha**		**51**	**28153**	**14.25%**
			Hypogastruridae	5	4866	2.46%
			Neanuridae	14	3273	1.66%

Phylum	Class	Order	Family	OTUs	Sequence reads	Relative sequence abundance (%)
			Odontellidae	1	396	0.20%
			Onychiuridae	29	19483	9.86%
			Tullbergiidae	1	103	0.05%
			unclassified_Poduromorpha	1	32	0.02%
		**Symphypleona**		**7**	**545**	**0.28%**
			Dicyrtomidae	2	250	0.13%
			Katiannidae	1	8	0.00%
			Sminthuridae	3	65	0.03%
			Sminthurididae	1	222	0.11%
		**unclassified_Collembola**		**9**	**156**	**0.08%**
			unclassified_Collembola	9	156	0.08%
	**Diplopoda**			**3**	**534**	**0.27%**
		**Polydesmida**		**2**	**529**	**0.27%**
			Cryptodesmidae	1	502	0.25%
			Paradoxosomatidae	1	27	0.01%
		**Polyxenida**		**1**	**5**	**0.00%**
			Polyxenidae	1	5	0.00%
	**Hexanauplia**			**1**	**9**	**0.00%**
		**Harpacticoida**		**1**	**9**	**0.00%**
			Canthocamptidae	1	9	0.00%
	**Insecta**			**237**	**53496**	**27.07%**
		**Blattodea**		**11**	**16641**	**8.42%**
			Pseudophyllodromiidae	1	3	0.00%
			Rhinotermitidae	7	10791	5.46%
			Termitidae	3	5847	2.96%

Phylum	Class	Order	Family	OTUs	Sequence reads	Relative sequence abundance (%)
		**Coleoptera**		**60**	**8465**	**4.28%**
			Cantharidae	2	11	0.01%
			Carabidae	4	850	0.43%
			Chrysomelidae	5	924	0.47%
			Coccinellidae	1	3	0.00%
			Corylophidae	1	3	0.00%
			Curculionidae	3	557	0.28%
			Dermestidae	1	58	0.03%
			Elateridae	17	2373	1.20%
			Hydraenidae	1	108	0.05%
			Latridiidae	8	177	0.09%
			Leiodidae	1	2	0.00%
			Scarabaeidae	1	2619	1.33%
			Silvanidae	1	231	0.12%
			Staphylinidae	5	345	0.17%
			Tenebrionidae	3	172	0.09%
			unclassified_Coleoptera	6	32	0.02%
		**Diptera**		**85**	**11486**	**5.81%**
			Agromyzidae	1	481	0.24%
			Anthomyiidae	1	1	0.00%
			Asilidae	1	28	0.01%
			Cecidomyiidae	28	1932	0.98%
			Ceratopogonidae	5	565	0.29%
			Chironomidae	14	982	0.50%
			Dolichopodidae	2	439	0.22%

Phylum	Class	Order	Family	OTUs	Sequence reads	Relative sequence abundance (%)
			Drosophilidae	2	773	0.39%
			Empididae	1	100	0.05%
			Keroplatidae	2	1619	0.82%
			Lauxaniidae	1	225	0.11%
			Mycetophilidae	2	33	0.02%
			Phoridae	6	746	0.38%
			Psychodidae	3	227	0.11%
			Scatopsidae	1	14	0.01%
			Sciaridae	8	323	0.16%
			Sphaeroceridae	2	737	0.37%
			Streblidae	1	15	0.01%
			Tephritidae	1	2	0.00%
			Tipulidae	1	115	0.06%
			unclassified_Diptera	2	2129	1.08%
		**Hemiptera**		**26**	**11464**	**5.80%**
			Achilidae	1	40	0.02%
			Aleyrodidae	1	7383	3.74%
			Aphididae	2	5	0.00%
			Cicadellidae	14	3822	1.93%
			Lygaeidae	1	20	0.01%
			Rhyparochromidae	2	179	0.09%
			Tingidae	5	15	0.01%
		**Hymenoptera**		**16**	**2393**	**1.21%**
			Eulophidae	1	3	0.00%
			Formicidae	10	2340	1.18%

Phylum	Class	Order	Family	OTUs	Sequence reads	Relative sequence abundance (%)
			Ichneumonidae	2	6	0.00%
			Platygastridae	1	6	0.00%
			Tenthredinidae	1	30	0.02%
			unclassified_Hymenoptera	1	8	0.00%
		**Lepidoptera**		**24**	**1594**	**0.81%**
			Blastobasidae	1	99	0.05%
			Bucculatricidae	1	239	0.12%
			Cosmopterigidae	2	27	0.01%
			Crambidae	4	97	0.05%
			Erebidae	1	386	0.20%
			Eriocottidae	1	70	0.04%
			Gelechiidae	2	29	0.01%
			Lecithoceridae	1	58	0.03%
			Meessiidae	1	1	0.00%
			Nymphalidae	1	3	0.00%
			Oecophoridae	1	29	0.01%
			Plutellidae	1	10	0.01%
			Pyralidae	2	415	0.21%
			Sesiidae	1	1	0.00%
			Tineidae	1	87	0.04%
			Tortricidae	1	31	0.02%
			unclassified_Lepidoptera	1	2	0.00%
			Xyloryctidae	1	10	0.01%
		**Odonata**		**2**	**19**	**0.01%**
			Coenagrionidae	1	2	0.00%

Phylum	Class	Order	Family	OTUs	Sequence reads	Relative sequence abundance (%)
			Macromiidae	1	17	0.01%
		**Psocoptera**		**7**	**291**	**0.15%**
			Amphipsocidae	2	32	0.02%
			Liposcelididae	5	259	0.13%
		**Thysanoptera**		**3**	**1130**	**0.57%**
			Thripidae	3	1130	0.57%
		**Trichoptera**		**3**	**13**	**0.01%**
			Hydropsychidae	3	13	0.01%
	**Protura**			**1**	**47**	**0.02%**
		**unclassified_Protura**		**1**	**47**	**0.02%**
			Eosentomidae	1	47	0.02%
	**Symphyla**			**3**	**4405**	**2.23%**
		**unclassified_Symphyla**		**3**	**4405**	**2.23%**
			Scolopendrellidae	3	4405	2.23%
	**unclassified_Arthropoda**			**9**	**1932**	**0.98%**
		**Diplura**		**8**	**1921**	**0.97%**
			Campodeidae	3	13	0.01%
			Japygidae	3	1882	0.95%
			Parajapygidae	2	26	0.01%
		**unclassified_Arthropoda**		**1**	**11**	**0.01%**
			unclassified_Arthropoda	1	11	0.01%

OTU, operational taxonomic unit, defined at 97% sequence similarity. Counts and relative sequence abundances are pooled across all samples. Relative sequence abundance was calculated as the number of reads assigned to a taxon divided by the total number of soil-associated arthropod reads across all samples, multiplied by 100. Control, uninjected plots; ATI1, a single injection in March 2024; ATI3, a single injection in March 2022.

Table S3. Family-level feeding-guild assignments for soil-associated arthropods and supporting references.

Class	Order	Family	Feeding guild	Reference
Arachnida	Araneae	Linyphiidae	Predators	[34]
Arachnida	Araneae	Salticidae	Predators	[35]
Arachnida	Araneae	Tetragnathidae	Predators	[35]
Arachnida	Araneae	Zodariidae	Predators	[36]
Arachnida	Mesostigmata	Ascidae	Predators	[37]
Arachnida	Mesostigmata	Macrochelidae	Predators	[38]
Arachnida	Mesostigmata	Melicharidae	Predators	[39]
Arachnida	Mesostigmata	Ologamasidae	Predators	[40]
Arachnida	Mesostigmata	Parasitidae	Predators	[41]
Arachnida	Mesostigmata	Parholaspididae	Predators	[42]
Arachnida	Pseudoscorpiones	Garypinidae	Predators	[43]
Arachnida	Pseudoscorpiones	Neobisiidae	Predators	[44]
Arachnida	Sarcoptiformes	Brachychthoniidae	Microbivores	[45]
Arachnida	Sarcoptiformes	Camisiidae	Microbivores	[46]
Arachnida	Sarcoptiformes	Ceratozetidae	Microbivores	[47]
Arachnida	Sarcoptiformes	Eremaeidae	Microbivores	[47]
Arachnida	Sarcoptiformes	Fortuyniidae	Microbivores	[48]
Arachnida	Sarcoptiformes	Galumnidae	Detritivores	[49]
Arachnida	Sarcoptiformes	Hypochthoniidae	Microbivores	[47]
Arachnida	Sarcoptiformes	Nothridae	Detritivores	[50]
Arachnida	Sarcoptiformes	Oppiidae	Microbivores	[51]
Arachnida	Sarcoptiformes	Scheloribatidae	Microbivores	[47]
Arachnida	Sarcoptiformes	Trhypochthoniidae	Detritivores	[52]
Arachnida	Trombidiformes	Bdellidae	Predators	[53]
Arachnida	Trombidiformes	Eupodidae	Microbivores	[40]
Arachnida	Trombidiformes	Rhagidiidae	Predators	[54]
Arachnida	Trombidiformes	Stigmaeidae	Predators	[55]
Collembola	Entomobryomorpha	Entomobryidae	Microbivores	[56]
Collembola	Entomobryomorpha	Isotomidae	Microbivores	[56]
Collembola	Entomobryomorpha	Tomoceridae	Microbivores	[56]
Collembola	Poduromorpha	Hypogastruridae	Microbivores	[56]
Collembola	Poduromorpha	Neanuridae	Microbivores	[57]
Collembola	Poduromorpha	Odontellidae	Detritivores	[58]
Collembola	Poduromorpha	Onychiuridae	Microbivores	[57]
Collembola	Symphypleona	Dicyrtomidae	Detritivores	[59]
Collembola	Symphypleona	Sminthurididae	Detritivores	[60]
Diplopoda	Polydesmida	Cryptodesmidae	Detritivores	[61]
Insecta	Blattodea	Rhinotermitidae	Herbivores	[62]

Class	Order	Family	Feeding guild	Reference
Insecta	Blattodea	Termitidae	Herbivores	[62]
Insecta	Coleoptera	Carabidae	Predators	[63]
Insecta	Coleoptera	Chrysomelidae	Herbivores	[64]
Insecta	Coleoptera	Curculionidae	Herbivores	[65]
Insecta	Coleoptera	Elateridae	Herbivores	[66]
Insecta	Coleoptera	Scarabaeidae	Herbivores	[67]
Insecta	Coleoptera	Silvanidae	Herbivores	[68]
Insecta	Coleoptera	Staphylinidae	Predators	[69]
Insecta	Diptera	Agromyzidae	Herbivores	[70]
Insecta	Diptera	Cecidomyiidae	Herbivores	[71]
Insecta	Diptera	Ceratopogonidae	Predators	[72]
Insecta	Diptera	Chironomidae	Detritivores	[71]
Insecta	Diptera	Dolichopodidae	Predators	[73]
Insecta	Diptera	Drosophilidae	Microbivores	[73]
Insecta	Diptera	Keroplatidae	Microbivores	[74]
Insecta	Diptera	Lauxaniidae	Detritivores	[75]
Insecta	Diptera	Phoridae	Predators	[73]
Insecta	Diptera	Psychodidae	Microbivores	[73]
Insecta	Diptera	Sciaridae	Microbivores	[76]
Insecta	Diptera	Sphaeroceridae	Detritivores	[77]
Insecta	Hemiptera	Aleyrodidae	Herbivores	[78]
Insecta	Hemiptera	Cicadellidae	Herbivores	[79]
Insecta	Hymenoptera	Formicidae	Predators	[80]
Insecta	Lepidoptera	Bucculatricidae	Herbivores	[81]
Insecta	Lepidoptera	Erebidae	Herbivores	[82]
Insecta	Lepidoptera	Pyralidae	Herbivores	[83]
Insecta	Psocoptera	Liposcelididae	Detritivores	[84]
Insecta	Thysanoptera	Thripidae	Herbivores	[85]
Symphyla	unclassified_Symphyla	Scolopendrellidae	Detritivores	[40]
Diplura	Diplura	Japygidae	Predators	[86]

Only families with an overall relative sequence abundance ≥ 0.10% were retained for feeding-guild assignment and subsequent guild-level analyses. Overall relative sequence abundance was calculated as the number of reads assigned to a family divided by the total number of reads across all samples in the corresponding assemblage, multiplied by 100. Complete taxonomic summaries and relative abundances are provided in Table S2 and are not repeated here. Reference numbers correspond to the reference list in the main manuscript.

Table S4. Family-level composition, numbers of operational taxonomic units (OTUs), and relative sequence abundance of Hymenoptera.

Family	OTUs	Sequence reads	Relative sequence abundance (%)
Ichneumonidae	162	3420	53.67%
Formicidae	5	462	7.25%
Vespidae	17	735	11.53%
Braconidae	56	659	10.34%
Colletidae	3	97	1.52%
Eulophidae	22	219	3.44%
unclassified_o__Hymenoptera	16	138	2.17%
Pompilidae	11	148	2.32%
Tenthredinidae	6	50	0.78%
Diapriidae	11	94	1.48%
Dryinidae	1	20	0.31%
Argidae	3	21	0.33%
Torymidae	5	26	0.41%
Pteromalidae	9	53	0.83%
Encyrtidae	3	24	0.38%
Chalcididae	3	22	0.35%
Platygastridae	5	39	0.61%
Eurytomidae	2	12	0.19%
Aphelinidae	4	16	0.25%
Bethylidae	7	30	0.47%
Apidae	3	14	0.22%
Halictidae	1	8	0.13%
Eucharitidae	2	11	0.17%
Megachilidae	1	8	0.13%
Gasteruptiidae	1	8	0.13%
Chrysididae	1	7	0.11%
Scelionidae	5	11	0.17%
Figitidae	2	4	0.06%
Trichogrammatidae	3	5	0.08%
Ormyridae	1	2	0.03%
Cynipidae	1	2	0.03%
Proctotrupidae	1	2	0.03%
Ceraphronidae	1	1	0.02%
Crabronidae	2	2	0.03%
Megastigmidae	1	1	0.02%

Family	OTUs	Sequence reads	Relative sequence abundance (%)
Eupelmidae	1	1	0.02%

OTU, operational taxonomic unit, defined at 97% sequence similarity. Counts and relative sequence abundances are pooled across all samples. Relative sequence abundance was calculated as the number of reads assigned to a taxon divided by the total number of Hymenoptera reads across all samples, multiplied by 100. Control, uninjected plots; ATI1, a single injection in March 2024; ATI3, a single injection in March 2022.

Table S5. Family-level feeding-guild assignments for Hymenoptera and supporting references.

Family	Feeding guild	Reference
Ichneumonidae	Parasitoids	[87]
Vespidae	Predators	[88]
Braconidae	Parasitoids	[88]
Formicidae	Predators	[89]
Eulophidae	Parasitoids	[88]
Pompilidae	Parasitoids	[88]
Colletidae	Pollinators	[90]
Diapriidae	Parasitoids	[91]
Pteromalidae	Parasitoids	[92]
Tenthredinidae	Herbivores	[93]
Platygastridae	Parasitoids	[88]
Bethylidae	Predators	[94]
Torymidae	Parasitoids	[95]
Encyrtidae	Parasitoids	[96]
Chalcididae	Parasitoids	[88]
Argidae	Herbivores	[97]
Dryinidae	Parasitoids	[88]
Aphelinidae	Parasitoids	[98]
Apidae	Pollinators	[99]
Eurytomidae	Parasitoids	[100]
Eucharitidae	Parasitoids	[101]
Scelionidae	Parasitoids	[88]
Halictidae	Pollinators	[102]
Megachilidae	Pollinators	[103]
Gasteruptiidae	Parasitoids	[104]
Chrysididae	Predators	[105]

Only families with an overall relative sequence abundance ≥ 0.10% were retained for feeding-guild assignment and subsequent guild-level analyses. Overall relative sequence abundance was calculated as the number of reads assigned to a family divided by the total number of reads across all samples in the corresponding assemblage, multiplied by 100. Complete taxonomic summaries and relative abundances are provided in Table S4 and are not repeated here. Reference numbers correspond to the reference list in the main manuscript.

References

34. Sunderland, K.D.; Fraser, A.M.; Dixon, A.F.G. Field and laboratory studies on money spiders (Linyphiidae) as predators of cereal aphids. *J. Appl. Ecol.* **1986**, *23*, 433–447.

35. Yamazaki, K.; Sugiura, S. Arthropods associated with bacterium galls on wisteria. *Appl. Entomol. Zool.* **2008**, *43*, 191–196.

36. Viel, N.; Wijacki, T.; Pétillon, J.; Pekár, S. Prevalence of two endosymbionts in range-expanding *Zodarion* spiders (Araneae: Zodariidae). *Biol. J. Linn. Soc.* **2025**, *144*, blae126. https://doi.org/10.1093/biolinnean/blae126.

37. Nielsen, P.S. Predation by *Blattisocius tarsalis* (Berlese) (Acari: Ascidae) on eggs of *Ephestia kuehniella* Zeller (Lepidoptera: Pyralidae). *J. Stored Prod. Res.* **2003**, *39*, 395–400.

38. Koehler, H.H. Mesostigmata (Gamasina, Uropodina), efficient predators in agroecosystems. *Agric. Ecosyst. Environ.* **1997**, *62*, 105–117.

39. Halliday, B. The enemy of my parasite is my friend: The possible role of predatory mites as biological control agents of pest beetles in soil. *Int. J. Acarol.* **2019**, *45*, 189–196.

40. Murray, A. A Chaos of Delight. 2020. Available online: https://www.chaosofdelight.org/.

41. Hrúzová, K.; Fenďa, P. The family Parasitidae (Acari: Mesostigmata)—History, current problems and challenges. *Acarologia* **2018**, *58 (Suppl.)*, 25–42.

42. Latifi, M.; Glida, H.; Torabi, A.; et al. *Holaspina* (syn. *Parholaspulus*) *persicum*, a new species of Parholospididae from Iran (Acari: Mesostigmata). *Acarologia* **2006**, *46*, 189–194.

43. Stanczak, N.; Harvey, M.S.; Harms, D.; Hammel, J.U.; Kotthoff, U.; Loria, S.F. A new pseudoscorpion genus (Garypinoidea: Garypinidae) from the Eocene supports extinction and range contraction in the European paleobiota. *PeerJ* **2023**, *11*, e15989. https://doi.org/10.7717/peerj.15989.

44. Koponen, S.; Sharkey, M.J. Northern records of *Microbisium brunneum* (Pseudoscorpionida, Neobisiidae) from eastern Canada. *J. Arachnol.* **1988**, *16*, 388–390.

45. McLean, M.A.; Parkinson, D. Introduction of the epigeic earthworm *Dendrobaena octaedra* changes the oribatid community and microarthropod abundances in a pine forest. *Soil Biol. Biochem.* **2000**, *32*, 1671–1681.

46. Hartenstein, R. Soil Oribatei V. Investigations on *Platynothrus peltifer* (Acarina: Camisiidae). *Ann. Entomol. Soc. Am.* **1962**, *55*, 709–713.

47. Schneider, K.; Renker, C.; Scheu, S.; Maraun, M. Feeding biology of oribatid mites: A minireview. *Phytophaga* **2004**, *14*, 247–256.

48. Pfingstl, T.; Kerschbaumer, M.; Shimano, S. Get a grip—Evolution of claw shape in relation to microhabitat use in intertidal arthropods (Acari, Oribatida). *PeerJ* **2020**, *8*, e8488.

49. Fernando, I.; Muhammad, F.N.; Furqoni, R.; Mario, M.B.; Puspitarini, R.D.; Trianti, I.; Syib'li, M.A.; Rizali, A. Local and landscape effects on the abundance and diversity of soil arthropods in citrus orchards of Malang and Batu, East Java, Indonesia. *Biodiversitas* **2024**, *25*, 3920–3927. https://doi.org/10.13057/biodiv/d251052.

50. Li, F.R.; Feng, Q.; Liu, J.L.; Sun, T.S.; Ren, W.; Guan, Z.H. Effects of the conversion of native vegetation to farmlands on soil microarthropod biodiversity and ecosystem functioning in a desert oasis. *Ecosystems* **2013**, *16*, 1364–1377. https://doi.org/10.1007/s10021-013-9689-5.

51. Seniczak, A.; Seniczak, S.; Słowikowska, M.; Paluszak, Z. The effect of different diet on life history parameters and growth of *Oppia denticulata* (Acari: Oribatida: Oppiidae). *Syst. Appl. Acarol.* **2017**, *22*, 749–758. https://doi.org/10.11158/saa.22.5.12.

52. Yoshida, T.; Hijii, N. The composition and abundance of microarthropod communities on arboreal litter in the canopy of *Cryptomeria japonica* trees. *J. For. Res.* **2005**, *10*, 35–42.

53. Hernandes, F.A.; Skvarla, M.J.; Fisher, J.R.; Dowling, A.P.G.; Ochoa, R.; Ueckermann, E.A.; Bauchan, G.R. Catalogue of snout mites (Acariformes: Bdellidae) of the world. *Zootaxa* **2016**, *4152*, 1–83. https://doi.org/10.11646/zootaxa.4152.1.1.

54. Zacharda, M.; Kučera, T. The Rhagidiidae (Acari: Prostigmata) in NW Lapland: Could their assemblages be climate warming monitors related to environmental and habitat patterns? *Pedobiologia* **2010**, *54*, 1–8.

55. Abdel-Khalek, A.; Momen, F. Cannibalism and intraguild predation of the predatory mite, *Agistemus exsertus* Gonzalez (Acari: Stigmaeidae). *Acta Phytopathol. Entomol. Hung.* **2013**, *48*, 259–268.

56. Gunn, A.; Cherrett, J.M. The exploitation of food resources by soil meso- and macroinvertebrates. *Pedobiologia* **1993**, *37*, 303–320.

57. Malmström, A.; Persson, T. Responses of Collembola and Protura to tree girdling—Some support for ectomycorrhizal feeding. *Soil Org.* **2011**, *83*, 279–285.

58. Checklist of the Collembola: Odontellidae. Available online: https://www.collembola.org/taxa/odontell.htm (accessed on 29 September 2026).
59. Cheng, H.J.; Janssens, F.; Chang, C.H. An updated checklist of Collembola in Taiwan, with DNA barcoding of *Papirioides jacobsoni* Folsom, 1924 (Symphypleona, Dicyrtomidae). *ZooKeys* **2022**, *1123*, 123–146.
60. Medeiros, G.d.S.; Nunes, R.C.; Zhang, F.; Godeiro, N.N.; Bellini, B.C. A new genus of Sminthurididae (Collembola, Symphypleona) from Brazil, with notes on the systematics of the family. *Diversity* **2022**, *14*, 960. https://doi.org/10.3390/d14110960.
61. Golovatch, S.I. Cave Diplopoda of southern China with reference to millipede diversity in Southeast Asia. *ZooKeys* **2015**, *510*, 79–94. https://doi.org/10.3897/zookeys.510.8640.
62. Bignell, D.E.; Roisin, Y.; Lo, N., Eds. *Biology of Termites: A Modern Synthesis*; Springer: Dordrecht, The Netherlands, 2011. https://doi.org/10.1007/978-90-481-3977-4.
63. BugGuide. Family Carabidae. Available online: https://bugguide.net/node/view/118#food.
64. North Carolina State University. Family Chrysomelidae. Available online: https://genent.cals.ncsu.edu/insect-identification/order-coleoptera/family-chrysomelidae/.
65. North Carolina State University. ENT 425 General Entomology: Order Coleoptera. Available online: https://genent.cals.ncsu.edu/insect-identification/order-coleoptera/.
66. ScienceDirect Topics. Elateridae. Available online: https://www.sciencedirect.com/topics/agricultural-and-biological-sciences/elateridae.
67. Karolyi, F.; Hansal, T.; Krenn, H.W.; Colville, J.F. Comparative morphology of the mouthparts of the megadiverse South African monkey beetles (Scarabaeidae: Hopliini): Feeding adaptations and guild structure. *PeerJ* **2016**, *4*, e1597. https://doi.org/10.7717/peerj.1597.
68. Weston, P.A.; Rattlingourd, P.L. Progeny production by *Tribolium castaneum* (Coleoptera: Tenebrionidae) and *Oryzaephilus surinamensis* (Coleoptera: Silvanidae) on maize previously infested by *Sitotroga cerealella* (Lepidoptera: Gelechiidae). *J. Econ. Entomol.* **2000**, *93*, 533–536.
69. Irmler, U.; Lipkow, E. Effect of environmental conditions on distribution patterns of rove beetles. In *Biology of Rove Beetles (Staphylinidae): Life History, Evolution, Ecology and Distribution*; Springer International Publishing: Cham, Switzerland, 2018; pp. 117–144.
70. Scheffer, S.J.; Winkler, I.S.; Wiegmann, B.M. Phylogenetic relationships within the leaf-mining flies (Diptera: Agromyzidae) inferred from sequence data from multiple genes. *Mol. Phylogenet. Evol.* **2007**, *42*, 756–775.
71. Marschall, L.A. *Flies: The Natural History and Diversity of Diptera*. 2012.
72. BugGuide. Family Ceratopogonidae. Available online: https://bugguide.net/node/view/19768#food.
73. Wiegmann, B.M. *Flies: The Natural History and Diversity of Diptera*. 2013.
74. Falaschi, R.L.; Amaral, D.T.; Santos, I.; Domingos, A.H.R.; Johnson, G.A.; Martins, A.G.S.; Viroomal, I.B.; Pompéia, S.L.; Mirza, J.D.; Oliveira, A.G.; et al. *Neoceroplatus betaryiensis* nov. sp. (Diptera: Keroplatidae) is the first record of a bioluminescent fungus-gnat in South America. *Sci. Rep.* **2019**, *9*, 11291. https://doi.org/10.1038/s41598-019-47753-w.
75. Miller, R.M. Ecology of Lauxaniidae (Diptera: Acalyptratae) 1. Old and new rearing records with biological notes and discussion. *Ann. Natal Mus.* **1977**, *23*, 215–238.
76. Dunham, A.E. Above and below ground impacts of terrestrial mammals and birds in a tropical forest. *Oikos* **2008**, *117*, 571–579.
77. Badenhorst, D. *The Bio-Ecology and Control of Coproica vagans and Coproica hirtula (Diptera: Sphaeroceridae) in Cattle Feedlots*. Thesis, University of the Free State, 1998.
78. Shah, M.M.R.; Liu, T.X. Feeding experience of *Bemisia tabaci* (Hemiptera: Aleyrodidae) affects their performance on different host plants. *PLoS ONE* **2013**, *8*, e77368.
79. Dominguez, E.; Murillo, V.; Orwat, J. Leafhopper food plants in a Neotropical forest in Panama (Hemiptera: Cicadellidae). *An. Acad. Bras. Cienc.* **2021**, *93*, e20190171.

80. Coleman, D.C.; Wall, D.H. Soil fauna: Occurrence, biodiversity, and roles in ecosystem function. In *Soil Microbiology, Ecology and Biochemistry*, 4th ed.; 2015; pp. 111–149.

81. Vargas, H.A.; Moreira, G.R.P. A new species of *Bucculatrix* Zeller (Lepidoptera: Bucculatricidae) associated with *Baccharis salicifolia* (Asteraceae) in northern Chile. *Zootaxa* **2012**, *3300*. https://doi.org/10.11646/zootaxa.3300.1.2.

82. Uyi, O.O.; Zachariades, C.; Marais, E.; Hill, M.P. Reduced mobility but high survival: Thermal tolerance and locomotor response of the specialist herbivore, *Pareuchaetes insulata* (Walker) (Lepidoptera: Erebidae), to low temperatures. *Bull. Entomol. Res.* **2017**, *107*, 448–457. https://doi.org/10.1017/S0007485316001103.

83. Stehr, F.W. Techniques for collecting, rearing, preserving, and studying immature insects. In *Immature Insects*; 1987; Volume 1, pp. 7–18.

84. Grimaldi, D.; Engel, M.S. Fossil Liposcelididae and the lice ages (Insecta: Psocodea). *Proc. R. Soc. B* **2006**, *273*, 625–633.

85. Diaz-Montano, J.; Fuchs, M.; Nault, B.A.; et al. Onion thrips (Thysanoptera: Thripidae): A global pest of increasing concern in onion. *J. Econ. Entomol.* **2011**, *104*, 1–13.

86. Sánchez-García, A.; Sendra, A.; Davis, S.; Grimaldi, D.A. Fossil diversity in 'dawn' hexapods (Diplura: Projapygoidea), with direct evidence for being chemically predaceous in the Cretaceous. *Zool. J. Linn. Soc.* **2023**, *198*, 847–870. https://doi.org/10.1093/zoolinnean/zlac101.

87. Gauld, I.D. Evolutionary patterns of host utilization by ichneumonoid parasitoids (Hymenoptera: Ichneumonidae and Braconidae). *Biol. J. Linn. Soc.* **1988**, *35*, 351–377.

88. He, J.H. *Hymenopteran Insect Fauna of Zhejiang*; Science Press, 2004.

89. Sakata, H. Density-dependent predation of the ant *Lasius niger* (Hymenoptera: Formicidae) on two attended aphids *Lachnus tropicalis* and *Myzocallis kuricola* (Homoptera: Aphididae). *Res. Popul. Ecol.* **1995**, *37*, 159–164.

90. Niu, Z.Q.; Kuhlmann, M.; Zhu, C.D. A review of the *Colletes succinctus*-group (Hymenoptera: Colletidae) from China with redescription of the male of *C. gigas*. *Zootaxa* **2013**, *3626*, 173–187.

91. Marchiori, C.H.; Oliviera, A.T.; Linhares, A.X. *Trichopria* sp. (Hymenoptera: Diapriidae) parasitoids of Diptera Muscoidea. *Arq. Inst. Biol.* **2000**, *67*, 131–133.

92. Aguilera, N.; Isaias, R.M.S.; Jorge, N.C.; Conejeros, M.J.; Becerra, J.; Nieves-Aldrey, J.L.; Guedes, L.M. Distinctive anatomical and histochemical responses of *Nothofagus obliqua* (Mirb.) Oerst (Nothofagaceae) to two galling Pteromalidae (Hymenoptera: Chalcidoidea) in Chile. *Flora* **2022**, *290*, 152056.

93. Price, P.; Roininen, H.; Ohgushi, T. Comparative plant-herbivore interactions involving willows and three gall-inducing sawfly species in the genus *Pontania* (Hymenoptera: Tenthredinidae). *Ecoscience* **1999**, *6*, 41–50.

94. Pérez-Lachaud, G.; Batchelor, T.P.; Hardy, I.C.W. Wasp eat wasp: Facultative hyperparasitism and intra-guild predation by bethylid wasps. *Biol. Control* **2004**, *30*, 149–155.

95. Trandem, N.; Westrum, K.; Hofsvang, T.; et al. Distribution and prolonged diapause of the rowan seed predators *Argyresthia conjugella* (Lepidoptera: Yponomeutidae) and *Megastigmus brevicaudis* (Hymenoptera: Torymidae) and their parasitoids in Norway. *Forests* **2023**, *14*, 847.

96. Hanks, L.M.; Gould, J.R.; Paine, T.D.; Millar, J.G.; Wang, Q. Biology and host relations of *Avetianella longoi* (Hymenoptera: Encyrtidae), an egg parasitoid of the eucalyptus longhorned borer (Coleoptera: Cerambycidae). *Ann. Entomol. Soc. Am.* **1995**, *88*, 666–671. https://doi.org/10.1093/aesa/88.5.666.

97. Endara, M.-J.; Nicholls, J.A.; Coley, P.D.; Forrister, D.L.; Younkin, G.C.; Dexter, K.G.; Kidner, C.A.; Pennington, R.T.; Stone, G.N.; Kursar, T.A. Tracking of host defenses and phylogeny during the radiation of neotropical *Inga*-feeding sawflies (Hymenoptera; Argidae). *Front. Plant Sci.* **2018**, *9*, 1237.

98. Li, Y.H.; Fan, Z.Y.; Zhu, Z.P.; et al. Effects of *Encarsia formosa* (Hymenoptera: Aphelinidae) parasitism on the feeding preference and performance of the whitefly predator *Serangium japonicum* (Coleoptera: Coccinellidae). *Crop Prot.* **2025**, *187*, 106959.

99. Dogterom, M.H.; Matteoni, J.A.; Plowright, R.C. Pollination of greenhouse tomatoes by the North American *Bombus vosnesenskii* (Hymenoptera: Apidae). *J. Econ. Entomol.* **1998**, *91*, 71–75.

100. Leggo, J.J.; Shorthouse, J.D. Modification of galls of *Diplolepis triforma* (Hymenoptera: Cynipidae) by the parasitoids *Eurytoma spongiosa* (Hymenoptera: Eurytomidae) and *Glyphomerus stigma* (Hymenoptera: Torymidae). *Can. Entomol.* **2006**, *138*, 681–696.

101. Hespenheide, H.A. Insect visitors to extrafloral nectaries of *Byttneria aculeata* (Sterculiaceae): Relative importance and roles. *Ecol. Entomol.* **1985**, *10*, 191–204.

102. Rodrigo Gómez, S.; Ornosa, C.; Selfa, J.; Guara, M.; Polidori, C. Small sweat bees (Hymenoptera: Halictidae) as potential major pollinators of melon (*Cucumis melo*) in the Mediterranean. *Entomol. Sci.* **2016**, *19*, 55–66. https://doi.org/10.1111/ens.12168.

103. Cripps, C.; Rust, R.W. Pollen foraging in a community of *Osmia* bees (Hymenoptera: Megachilidae). *Environ. Entomol.* **1989**, *18*, 582–589.

104. Austin, A.D. The importance of "species" in biodiversity studies: Lessons from a mega-diverse group—the parasitic Hymenoptera. In *The Other 99%: The Conservation and Biodiversity of Invertebrates*; 1999; pp. 159–165.

105. Būda, V.; Radžiutė, S.; Apšegaitė, V.; Budrys, E.; Budrienė, A.; Blažytė-Čereškienė, L. Cuticular hydrocarbons as putative sex pheromones and mating peculiarity of cuckoo wasps *Chrysis angustula*, *Chrysis fulgida* and *Chrysis iris* (Hymenoptera: Chrysididae). *Arch. Insect Biochem. Physiol.* **2024**, *117*, e70014. https://doi.org/10.1002/arch.70014.
